# Interactions between Hepatitis C Virus and the Human Apolipoprotein H Acute Phase Protein: A Tool for a Sensitive Detection of the Virus

**DOI:** 10.1371/journal.pone.0140900

**Published:** 2015-10-26

**Authors:** Ilias Stefas, Sylvia Tigrett, Grégor Dubois, Marco Kaiser, Estelle Lucarz, Delphine Gobby, Dorothy Bray, Heinz Ellerbrok, Jean Pierre Zarski, Francisco Veas

**Affiliations:** 1 ApoH-Technologies, Faculté de Pharmacie, Université de Montpellier, Montpellier, France; 2 Institut de Recherche pour le Développement, UMR-Ministère de la Défense 3, Laboratoire d’Immuno-Physiopathologie Moléculaire Comparée, Faculté de Pharmacie, Montpellier, France; 3 GenExpress GmbH, Berlin, Germany; 4 Immunoclin Corporation, Washington, DC, United States of America; 5 Robert Koch-Institute, Centre for Biological Threats and Special Pathogens, Highly Pathogenic Viruses, Berlin, Germany; 6 Clinique d’Hépato-gastroentérologie, Centre Hospitalier Universitaire de Grenoble, IAB, INSERM U823, Grenoble, France; Saint Louis University, UNITED STATES

## Abstract

The Hepatitis C virus (HCV) infection exhibits a high global prevalence frequently associated with hepatocellular carcinoma, taking years to develop. Despite the standardization of highly sensitive HCV quantitative RT-PCR (qRT-PCR) detection methods, false-negative diagnoses may be generated with current methods, mainly due to the presence of PCR inhibitors and/or low viral loads in the patient’s sample. These false-negative diagnoses impact both public health systems, in developing countries, and an in lesser extent, in developed countries, including both the risk of virus transmission during organ transplantation and/or blood transfusion and the quality of the antiviral treatment monitoring. To adopt an appropriate therapeutic strategy to improve the patient’s prognosis, it is urgent to increase the HCV detection sensitivity. Based upon previous studies on HBV, we worked on the capacity of the scavenger acute phase protein, Apolipoprotein H (ApoH) to interact with HCV. Using different approaches, including immunoassays, antibody-inhibition, oxidation, ultracentrifugation, electron microscopy and RT-PCR analyses, we demonstrated specific interactions between HCV particles and ApoH. Moreover, when using a two-step HCV detection process, including capture of HCV by ApoH-coated nanomagnetic beads and a home-made real-time HCV-RT-PCR, we confirmed the presence of HCV for all samples from a clinical collection of HCV-seropositive patients exhibiting an RT-PCR COBAS® TaqMan® HCV Test, v2.0 (COBAS)-positive result. In contrast, for HCV-seropositive patients with either low HCV-load as determined with COBAS or exhibiting HCV-negative COBAS results, the addition of the two-step ApoH-HCV-capture and HCV-detection process was able to increase the sensitivity of HCV detection or more interestingly, detect in a genotype sequence-independent manner, a high-proportion (44%) of HCV/RNA-positive among the COBAS HCV-negative patients. Thus, the immune interaction between ApoH and HCV could be used as a sample preparation tool to enrich and/or cleanse HCV patient’s samples to enhance the detection sensitivity of HCV and therefore significantly reduce the numbers of false-negative HCV diagnosis results.

## Introduction

Until the recent introduction of hepatitis C virus (HCV) screening tests, this viral infection has represented the major cause of blood transfusion-associated hepatitis [1]. Near 170 million people worldwide are infected with HCV [2], a prevalence about four-fold higher than that of HIV. More than 70% of the HCV-infected individuals develop a chronic infection considered as a major cause of liver cirrhosis and hepatocellular carcinoma [3]. Other lympho-proliferative disorders may also be associated with HCV infection, including mixed cryoglobulinemia (MC) and Non-Hodgkin’s lymphoma (NHL) [4]. Although the HCV pathogenesis is not well understood, viral infection progresses slowly and often ends in chronic diseases. HCV mainly targets the liver cells [5], but this virus may also replicate in extra-hepatic cells such as T, B and monocyte cell subsets from chronically infected individuals [6].

HCV is a small enveloped, positive strand RNA virus belonging to the *Hepacivirus* genus from the *Flaviviridae* family [7]. Based upon the sequence heterogeneity of its genome, HCV is classified into six major genotypes and more than 100 subtypes [8]. Its genome of approximately 9 600 nucleotides encodes a polyprotein precursor of about 3 000 amino acids. This viral polyprotein is cleaved by both viral and host proteases to generate mature structural proteins, including the capsid and two glycosylated envelope proteins (E1 and E2), as well as non structural proteins. Reliable *in vitro* propagation systems are pending, infectious HCV virions have not yet been isolated and functionally characterized. Heterogeneous viral populations from human sera have been reported, including defective particles [9], such as non-enveloped nucleocapsids [10, 11] as well as virions bound to either immunoglobulins or serum β lipoproteins [12–14].

Although the whole process of HCV cell entry mechanisms remains unclear, several lines of evidence show that the HCV envelope interacting with cell surface proteins is involved in the initiation of infection by mediating virus-host cell membrane interaction [15]. Thus, it has been reported that cell surface heparan sulphates [16] and proteins including the tetraspanin CD81 [17], the scavenger receptor class B type 1 [18], the LDL receptor [19] and the asialoglycoprotein receptor [20] could mediate the E2 binding and subsequent HCV internalization. However, as most of the studies on HCV cell entry are generated by *in vitro* models, it is still unclear whether any of the prior cited molecules could act as a functional receptor on human hepatocytes [21].

HCV/RNA-containing particles exhibit highly heterogeneous densities [22, 23]. The particles corresponding to different fractions yielded after gradient-density centrifugation could be completely, partially or not at all co-precipitated with an anti-beta lipoprotein serum [12]. Consequently, this observation suggests an association between the virus with plasma lipoproteins, including LDL, VLDL [14] and HDL [24–26]. The lipoproteins particles are complex aggregates of lipids (mainly triglycerides, phospholipids and cholesterol) and proteins (apolipoproteins). A recent study [27] has shown that the serum VLDL-TG/non-VLDL-TG ratio, which focused on TG metabolic alterations, may be an early indicator of HCV-related chronic hepatitis. Among the apolipoproteins, ApoA-I, ApoB, ApoC-I and ApoE are involved in the infectivity, production and transport of HCV [28–33]. This association has been correlated with both the virus infectivity and genotype. However, although these lipoproteins and apolipoproteins are required for HCV infectivity and production, they are not HCV-specific, since they interact with other viruses. Thus, ApoA-I interacts with dengue and hepatitis B viruses [34, 35]. VLDL, HDL and LDL interact with rotavirus, dengue and herpes simplex viruses [36–38].

Apoliporotein H (ApoH), also known as β_2_–glycoprotein I (β_2_–GPI), has been primarily described as a mitochondrial agglutinin, exhibiting a strong affinity for negatively-charged phospholipids [39]. A phospholipid-binding site, located in the fifth domain of ApoH has been identified [40, 41]. It has been clearly demonstrated that HCV from human-infected plasma co-elutes with VLDL. This association with host’s lipoproteins may explain the low buoyant density of HCV (**<**1.10 g/mL). This reported observation was associated with the highest infectious material [42]. Because several lines of evidence suggest that HCV circulates in the bloodstream as a hybrid lipoviral particle, so called LVP [43], it is possible to hypothesize that ApoH could bind to HCV particles through anionic phospholipids of the viral envelope. We have previously reported the association of ApoH with hepatitis B virus (HBV) human immunodeficiency virus (HIV) and rotavirus [44–46]. Altogether, these results led us to investigate whether the ApoH, a protein partially associated with the plasma lipoprotein fraction [39], has the capacity to bind to HCV particles. The earliest detection of viruses is a crucial step to establish an individual accurate diagnosis of the disease, to engage an appropriate therapeutic management (including drug monitoring efficiency) leading to improved prognostic for the patient. This crucial step is also necessary in public health to set up the appropriate countermeasures for both control and/or prevention of the dissemination of the virus, particularly in developing countries. Despite the development of HCV quantitative RT-PCR and derived methods to detect and quantify the viral load in patients, HCV occult infections have been reported [47, 48]. An occult HCV infection is mainly characterized by the presence of HCV/RNA in liver cells or in peripheral blood mononuclear cells, but with negative detection of HCV/RNA in serum using current tests, in the absence or the presence of anti-HCV antibodies. A recent Italian cohort study evidenced that HCV occult infections may occur in populations selected for not having any hepatic disease. These studies concluded that a potential risk of infectious HCV spreading should be considered [49], in particular during organ transplantation or blood transfusion, mainly in developing countries with poor diagnostic settings [50]. In Egypt, regular sexual unprotected intercourses between HCV-positive patients and HCV-negative spouses results in a prevalence about 4% of HCV occult infections among the previous HCV-negative individual partners [51]. In addition, recent investigations on organ transplant have identified an increased risk of HCV transmission by organ donors harbouring very recent HCV infection with negative nucleic acid testing to naive individuals [52]. These facts point out the urgent need for innovative methods to further improve the HCV diagnostics and overcome any risk of residual viral loads.

Since HCV circulates in the bloodstream as an enveloped viral particle and based on the capacity of ApoH to bind some anionic phospholipids, we hypothesized that ApoH binds HCV, and that this binding is done through phospholipids from the HCV envelope. This study mainly demonstrates the capacity of ApoH to bind HCV, which ultimately allowed the capture and concentration of HCV from human fluids (sera or plasma) thus enhancing the detection sensitivity of the HCV molecular diagnostics. To this purpose, specific capture tools have been developed, including ApoH-coated solid supports such as nanomagnetic beads and ELISA immunoassay on microtitration plates.

## Materials and Methods

### Reagents

Bovine serum albumin (BSA) was purchased from Fluka (Buchs, Switzerland), 20% human albumin solutions were obtained from the Etablissement Français du Sang in Montpellier, France and α1-acid glycoprotein was purchased from Sigma (St. Louis, Mo, USA). Maxisorp microtiter plates (96-well) were supplied by NUNC (Roskilde, Denmark). The following monoclonal and polyclonal antibodies were used: the mouse anti-human ApoH, 8C3, MAb was kindly donated by J. Arvieux [53]), the mouse anti-human thyroglobulin 2, TG2, MAb was a kind gift from Sanofi, Montpellier, France, the mouse anti-HCV/E2, 3A2C11, MAb was kindly provided by BioMérieux, (Lyon, France) and a goat peroxidase-conjugated anti-mouse serum was purchased from Sigma (St. Louis, Missouri, USA). The DNA molecular size marker 1 Kb Plus DNA Ladder was purchased at Life Technologies (Saint Aubain, France). Nucleic acids sequencing was carried out by MilleGen (Toulouse, France). ApoH was purified from human plasmatic albumin solutions as previously described [45].

### Serum samples

Serum samples from healthy blood donors and sera from HCV-infected patients with chronic hepatitis were obtained from the following laboratories: Etablissement Français du Sang of Montpellier, France; the Virology Laboratories of the Centre Hospitalier Universitaire of Grenoble, France. The presence of anti-HCV antibodies and HCV-RNA in hepatitis patient’s sera were respectively established by using both ELISA (ELISA 3, Ortho Diagnostic Systems, Raritan, NJ, USA) and real-time HCV RT-PCR with a limit of detection comprised between 9.3 and 20 IU/mL (COBAS® TaqMan® HCV Test, v2.0, Roche Diagnostics, Basel, Switzerland).

### HCV capture by ApoH-coated nanomagnetic beads and viral RT-PCR detection

A total of 10 μL of ApoH-coated nanomagnetic bead suspension (ApoH-Technologies, Montpellier, France) was added to 100 μL of serum diluted in an acetate acidic buffer. The mixture was incubated at 6°C for 30 min on an Eppendorf Thermomixer Comfort (Lyon, France). The tube was placed against a magnet for four minutes and the unbound serum fraction was removed. The beads were then resuspended in 560 μL of lysis buffer (AVL from the QIAamp^®^ Viral RNA Mini Kit, Qiagen, Germany) containing 8 units of RNase OUT (Life Technologies, Carlsbad, USA) and 5.6 μg RNA carrier and heated to 35°C for 10 min. The supernatant was separated from the beads by retaining them with a magnet. The viral nucleic acids in the supernatant were purified according to the procedure described in the QIAamp^®^ Viral RNA Mini Kit (QIAGEN, Germany).

We created an “open” home-made HCV-reverse transcription and polymerase chain reaction (RT-PCR) that was carried out by using KY80 primers (sense: 5′-GCAGAAAGCGTCTAGCCATGGCGT-3′) and KY78 (antisense: 5′-CTCGCAAGCACCCTATCAGGCAGT-3′) leading to the amplification of a sequence of 244 nucleotides within the conserved 5′NC region of the HCV genome [54]. For end-point PCR and in-gel results, the following protocol was applied. HCV/RNA was incubated with the primers for 7 min at 70°C to linearize the RNA and cooled on ice. RT was allowed to proceed for 15 min at 50°C and was followed by 15 min incubation at 95°C to inactivate the reverse transcriptase, activate the polymerase and facilitate denaturation of RNA-DNA heteroduplexes. PCR amplification proceeded with 40 cycles at 94°C for 60 s, 55°C for 30 s and 72°C for 60 s and a final extension step at 68°C for 7 min. The RT-PCR mixture was carried out by using QIAGEN® OneStep RT-PCR Kit, in accordance the manufacturer's procedure (Qiagen, Germany).

For the “open” home-made quantitative HCV RT-PCR (qRT-PCR), the following protocol was applied. HCV/RNA was incubated with random N6 primers for 6 min at 65°C to linearize the RNA and cooled on ice. Primer annealing was performed for 10 min at 25°C. RT was allowed to proceed for 50 min at 37°C and was followed by 15 min incubation at 70°C (according to Life Technologies™ procedure M-MLV RT kit). The cDNA obtained was 10-fold diluted and amplified in a 480 LightCycler with the SybrGreen I master mix including KY78 and KY80 primers (Roche™, Basel, Switzerland). After 5 min denaturation at 95°C, 45 cycles were performed: 10 s at 95°C, 10 s at 60°C, 10 s at 72°C and fluorescence was measured in each cycle during extension. The DNA was heated to 95°C for 5 min and cooled to 65°C for 1 min before measuring the fluorescence during a final melting curve from 65°C to 97°C. An HCV plasmid (pGEM-T easy, Promega, with an HCV/PCR-insert) was used as template to make the standard curve and compute HCV copies.

### Enzyme immunoassay using ApoH for the HCV capture

ApoH-coated microtiter plates (96-well) were saturated with 200 μL of 3% BSA in Tris 50 mM, pH 9.0, NaCl 0.15 M, for 1 h at 37°C and 4 washes with 10 mM phosphate buffer, pH 7.2, 0.15 M NaCl (PBS) containing 0.005% of Tween 20 (Sigma, St. Louis, Mo, USA). One hundred microliters of serum or viral preparations from CsCl gradient separation diluted in a Tris 50 mM, pH 7.6 buffer were added per well. After 1 h incubation at 37°C followed by 4 washings with PBS, 100 μ L of anti-HCV or irrelevant MAb was added and incubated at 37°C for 1 h. Subsequently, the wells were washed 4 times with PBS and 100 μL of peroxidase-conjugated polyclonal anti-mouse serum were added. Finally, after 1 h incubation at 37°C, the wells were washed 6-times with PBS and O-Phenylene-Diamine (OPD, Sigma, St. Louis, Missouri, USA) was added according to the manufacturer's procedure. Optical density was measured at 492 nm with a Titertek Multiskan Plus spectrophotometer (Flow Laboratories, USA). The data were expressed as a P/N ratio (mean ratio ± standard deviation, SD) which represents the mean of four replicate absorbance values of the sample (P) as compared to the mean absorbance value of at least 5 negative control sera (N).

### Gradient centrifugation of serum

To purify HCV particles from serum, 10 mL of pooled serum samples from either 20 HCV-infected patients or 20 healthy carriers were used for this study. After filtration through two layers of gauze, serum was centrifuged without adjustment of density for 1 h at 436,000 x g at 4°C in a TLA-120.2 fixed angle rotor (Beckman, Fullerton, CA). After centrifugation, three fractions were respectively recovered: the yellow cake (VLDL) phase located over the supernatant, the supernatant and the pellet. The VLDL fraction was resuspended in 0.15 M NaCl solution and the centrifugation was repeated twice and stored for further analyses. The pellet was suspended in PBS buffer and re-centrifuged twice under the same conditions. The final pellet was resuspended in a final PBS volume corresponding to a 1/10 of the initial serum volume. One hundred microliter portions of these HCVp concentrates were layered onto 900 μL of either a sucrose or a CsCl gradient [10–60% (w/w)] and centrifuged for 18 h at 300,000 x g at 4°C.

### Plasma lipoprotein separation

Duplicate samples of freshly-collected EDTA-plasma were used to separate the three major lipoprotein classes by sequential, isopycnic centrifugation in a TLA-120.2 fixed angle rotor (Beckman, Fullerton, CA), as described previously [55]. Density (r) adjustments were made with stock solutions of KBr in 0.15 M NaCl and four fractions were obtained: very-low density lipoproteins (VLDL; r <1.006 g/L), low-density lipoproteins (LDL; r: 1.006–1.063 g/L), high-density lipoproteins (HDL; r: 1.063–1.21 g/L), and very-high-density lipoproteins (r>1.21 g/L, which also include bulk plasma proteins). Each fraction was collected, extensively dialyzed against 150 mM NaCl/ 0.24 mM EDTA pH 7.4 at 4°C and filtered through 0.22 μm-pore size filters (Millipore S.A., St Quentin, France).

### Electron microscopy

Samples of HC-VP (Hepatitis C virus particles) isolated from sucrose gradient were incubated in 2% glutaraldehyde in PBS and applied to an ApoH-coated400-mesh Formvar-carbon-coated copper grid for approximately 2 min. Excess fluid was drawn away with filter paper. The sample was then negatively stained with 1% (w/v) filtered aqueous uranyl acetate for 2 min and, after washing in 1% uranyl acetate, examined using a Zeiss EM 10C/CR transmission electron microscope, as described previously [44].

### Statistical analyses

Bland-Altman plot analysis was performed to assess the agreement level between the quantification of HCV/RNA with and without ApoH-sample pretreatment method. ANOVA multiparametric tests have been done using the GraphPad PRISM 5 software™.

### Ethic statements

An archived collection of sera from HCV-seropositive human patients with or without a viral load detection determined by a quantitative real-time PCR technology using the COBAS® TaqMan® HCV Test, v2.0 were used for this study (Metropolitan French USDEP project) and were part of an already-established collection "HCV-LO-2005-2006" of anonymized leftover samples at the Virology Lab of The University Hospital Center (CHU) “La Tronche”, Grenoble, France. The shipping of these clinical samples for research purposes to the Laboratoire d’Immuno-Physiopathologie Moléculaire Comparée (Montpellier, France) during 2005 and 2006, were perfectly in line with the French ethical regulations. Healthy donors samples and human albumin was purchased at the Centre Regional de Transfusion Sanguine de Lille, France.

## Results

### Capture of serum HC-VP by ApoH

Two different approaches were used to evidence the binding between ApoH and HCV-related particles: an RT-PCR assay, in order to detect viral RNA after the virus capture with ApoH-coated beads and an immuno-enzymatic assay in order to detect ApoH-captured HCV antigens, using ApoH-coated plates.

**Fig 1:**
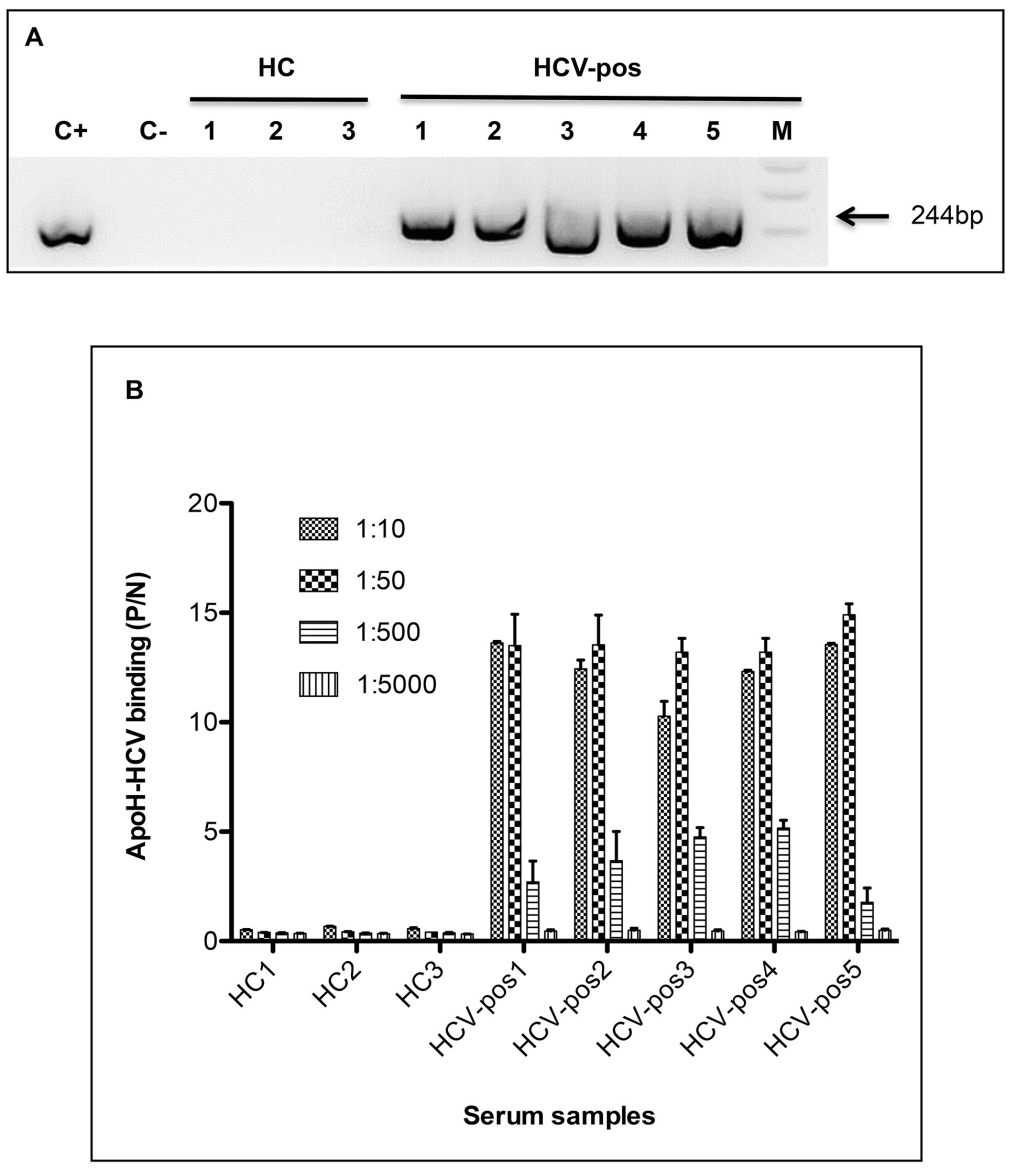
Binding between ApoH and Hepatitis C virus-related particles. Sera from both five HCV-positive patients (HCV-pos) and three HCV-negative healthy controls (HC) were assayed for viral detection. (A) RT-PCR detection of HCV from 20-fold diluted sera, after the viral capture by ApoH-coated beads. Lanes M, C+ and C- respectively correspond to the DNA molecular mass marker (1 Kb Plus DNA Ladder), the positive control (pGEM-T easy with an HCV/PCR-insert) and the negative extraction control. (B) ApoH-ELISA immunoassay to detect HCV-related particles binding from serially-diluted sera. Aliquots from the same sera as used for RT-PCR were 10, 50, 500, and 5,000-fold diluted and subsequently detected with the anti-HCV/E2 MAb.

In the experiment shown in Fig 1A, sera from both five HCV-infected patients exhibiting chronic hepatitis and three healthy blood donors were diluted 20-fold in an acetate buffer, and subsequently added to ApoH-coated nanomagnetic beads. The captured HCV were detected by RT-PCR. HCV/RNA was detected in all five patients, whereas no PCR amplicon band was detected for the healthy blood donors’ sera.

In the experiment shown in Fig 1B, aliquots from the same sera used in Fig 1A were diluted in Tris-buffer and then added to ApoH-coated microtitration plates. Then captured HCV envelope antigens were detected with the anti-HCV/E2 MAb. Thus, ApoH captured E2-related antigens from each one of the HCV-positive patients’ sera. The mean of P/N ratios for the three healthy donors was 0.79 ± 0.07. All five HCV-positive sera exhibited significant P/N values (p<0.0001) at the 10-fold and 50-fold dilutions by an immuno-enzymatic assay. Therefore, we used these optimal dilutions for our ApoH-binding studies by the ApoH immunoassay. Anti-HCV/core and anti-HCV/E2 MAbs (data not shown) were assayed to detect the ApoH–HCV interaction. Only the anti-HCV/E2 MAb gave a significant signal after the serum incubation with the ApoH-coated ELISA plate. No signal was observed with the anti-HCV/core MAb, suggesting that the presence of whole enveloped virus is necessary to obtain a binding of HCV with ApoH. These results indicate that ApoH was able to capture HCV particles from patient’s sera. The simultaneous recognition by an anti-HCV/E2 MAb and the presence of viral RNA suggests that ApoH is able to capture whole HCV particles.

**Fig 2:**
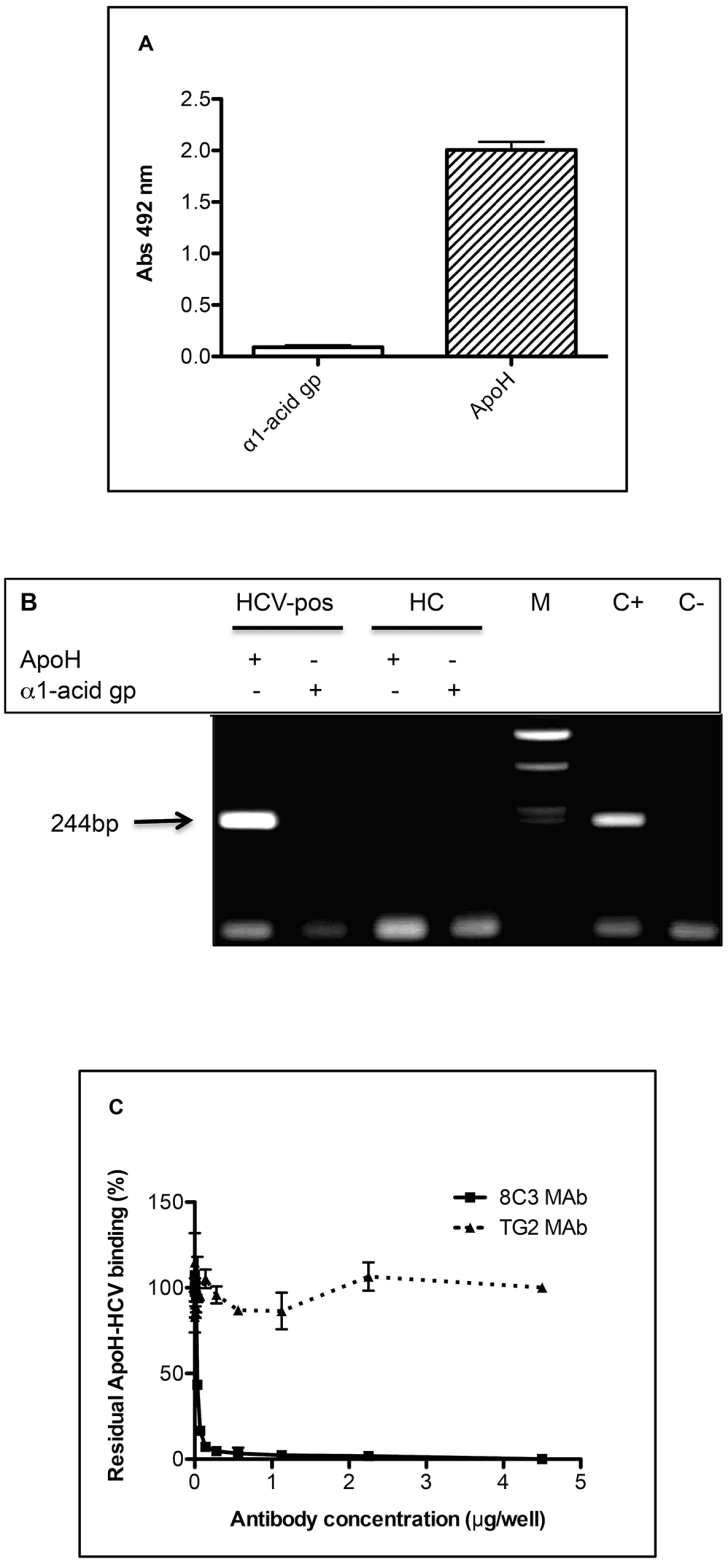
Binding specificity of ApoH and HCV. (A) One hundred microliters of sera from five HCV-positive patients were incubated with either the α1-acid glycoprotein- or the ApoH- coated plates. The HCV-related antigens were revealed with the anti-HCV/E2 MAb. The bars represent the corresponding means with SD. (B) Sera from both a single healthy control (HC) and one HCV-pos were either incubated with α1-acid glycoprotein or with the ApoH-coated magnetic beads and HCV/RNA was subsequently detected by RT-PCR. M, C+ and C- respectively correspond to the DNA molecular mass marker (1 Kb Plus DNA Ladder), the positive control (plasmid pGEM-T easy with an HCV/PCR-insert) and the negative PCR control. (C) The inhibition of the interaction between ApoH and HCV, previously shown, was assessed either by using the anti-ApoH, 8C3, MAb, or the anti-thyroglobulin, TG2, irrelevant MAb. Increasing concentrations of either 8C3 (solid line) or TG2 (hatched line) were used for the pre-incubation of the ApoH-coated plate with a 50-fold diluted HCV-positive serum.

To assess the involvement and specificity of coated ApoH to capture HCV from patients’ sera, plates and nanomagnetic beads were coated with another serum acute phase protein, the α1-acid glycoprotein, the binding was measured by both immunoassay (with an anti-HCV/E2 MAb) and HCV RT-PCR. Both Fig 2A and 2B showed that the α1-acid glycoprotein was not able to capture HCV as compared with ApoH-coated supports.

In order to assess the specificity of interaction between ApoH and HCV, we used ApoH-coated plates to capture HCV and we assessed the inhibition of viral capture using an anti-ApoH MAb (Fig 2C). An irrelevant non-specific antibody directed against the thyroglobulin protein was used as control. Indeed, only the mouse anti-ApoH, 8C3, MAb strongly and specifically inhibited the interaction between the ApoH-coated well plate and the HCV envelope. In contrast, the irrelevant mouse anti-tyroglobulin, TG2, MAb used control, didn’t show any effect on the binding between ApoH and HCV particles. These observations clearly indicate that HCV capture was done in an ApoH-dependent manner.

To ascertain the specificity of these results as well as the efficiency of the HCV-capture by ApoH, a broad range of sera coming from either HCV-seropositive or HCV-seronegative patients, exhibiting different pathologies, was assayed for the detection of HCV by the ApoH immunoassay. Table 1 shows that only sera exhibiting a COBAS HCV-RT-PCR positive result were also HCV-positive with the ApoH ELISA immunoassay.

**Table 1:** Detection of HCV antigen with the HCV-ApoH immunoassay on patients’ sera exhibiting different infections.

Serum samples	Negative ApoH-immunoassay	Positive ApoH-immunoassay	Positive COBAS HCV-RT-PCR
Healthy controls, blood donors (n = 100)	100	0	0
Autoimmune diseases (n = 20)	20	0	0
Multiple Sclerosis (n = 15)	15	0	0
HIV/AIDS (n = 10)	9	1^a^	1
HAV (n = 10)	10	0	0
HBV (n = 20)	15	5 ^b^	5
HCV–seropositive (n = 120)	6 ^c^	114	114

^a^HCV- HIV co-infected patients

^b^HCV- HBV co-infected patients

^c^ These samples also exhibited a negative RT-PCR COBAS® TaqMan® HCV Test, v2.0- result.

### Influence of HCV genotype on virus binding with ApoH

Sera of 43 hospital-archived clinical samples from HCV seropositive patients, harboring the HCV genotypes 1 to 5, as well as 10 sera from healthy blood donors were diluted in a Tris buffer and then added to ApoH-coated microtitration plates. Once captured, the HCV envelope antigen was detected using an anti-HCV/E2 MAb. As shown in Table 2, no significant differences between HCV genotype and ApoH-binding to HCV-related particles have been found. All the 10 sera from healthy blood donors were confirmed to be HCV-negative and the mean of P/N ratio was 0.49 ± 0.08. Two HCV patients were not detected by the ApoH ELISA immunoassay. One of these negative samples counted among the 11 that were previously diagnosed as HCV genotype 1; the second negative sample counted among the three that were diagnosed as HCV genotype 5. The absence of HCV detection signal was probably due to the lower sensitivity of the ApoH immuno-enzymatic assay as compared with the higher sensitivity of the RT-PCR format. The P/N ratios of HCV-genotype 1 to 5 from patients’ sera exhibited significant values and were respectively of 1.78 ± 0.57, 1.90 ± 0.45, 2.69 ± 1.29, 1.80 ± 0.35 and 1.7 ± 0.25.

**Table 2:** ApoH-immunoassay on different sera from patients harbouring different HCV genotypes.

HCV-positive patients	HCV-1 ^a^	HCV-2 ^a^	HCV-3 ^a^	HCV-4 ^a^	HCV-5 ^a^	TOTAL of positive samples
HCV/RNA detection using COBAS HCV RT-PCR (without ApoH)	11	9	10	10	3	43
HCV/E2 detection using the ApoH-immunoassay	10^b^	9	10	10	2^c^	41
HCV detection in healthy controls	0	0	0	0	0	0

^a,^ corresponding HCV genotype

^b, c^ This test didn’t detect one COBAS RT-PCR HCV-positive sample.

### Ultracentrifugation and density distribution of ApoH-captured HCV particles

HCV-positive sera submitted to buoyant and sedimentation processes exhibited a high viral heterogeneity in their gradient location. This heterogeneity may be due to the existence of defective particles or the association of viral particles to different serum components, including antibodies, low-density lipoprotein (LDL), very low-density lipoprotein (VLDL) and high-density lipoprotein (HDL).

In order to assess whether the binding of ApoH to HCV was able to retain whole HCV/RNA particles, a pool of HCV/RNA-positive sera from untreated patients or HCV-negative blood donors sera were spun by ultra-centrifugation at 436,000 x g. The resulting pellets, supernatants and floating lipids were tested for their capacity to bind to ApoH-coated microtitration plates and subsequently tested for their RNA content by RT-PCR. Table 3 shows that the HCV-related antigens are found in both the VLDL as well as in the supernatant fractions of the centrifuged sera. These results confirm the significant heterogeneity of distribution for hepatitis C related particles. Table 3 also shows a significant lower signal for the pellet fraction. Comparatively, after centrifugation of a pool of HBV-DNA positive sera, the fraction density distribution of ApoH-captured HBV/HBsAg was different from the ApoH-captured HCV/E2. Almost the totality of the ApoH-captured HBV/HBsAg was found in the pellet. In addition, we have not detected either HCV/E2 or HBV/HBsAg from healthy blood donor samples after ultracentrifugation (data not shown).

**Table 3:** Detection of HCV and HBV within different centrifuged serum fractions using the appropriate ApoH-immunoassay.

Viral detection in different serum^a^ fractions	HCV/E2 detection (P/N)	HBV/HBsAg detection (P/N)
Serum before centrifugation	11.20 ± 6.43	34.30 ± 6.67
Floating lipids (VLDL)	12.54 ± 7.01	2.00 ± 1.19
Serum after centrifugation	10.62 ± 5.37	9.70 ± 5.41
Pellet	6.66 ± 1.44	50.80 ± 3.34

^a^ Sera from patients with chronic hepatitis C or B were ultracentrifuged for 1 h at 436,000 x g and subsequently tested for HCV or HBV using the corresponding virus-ApoH immunoassay. The presence of viral antigens was revealed using either anti-E2 or anti-HBsAg MAbs. Results are expressed as P/N values.

**Fig 3:**
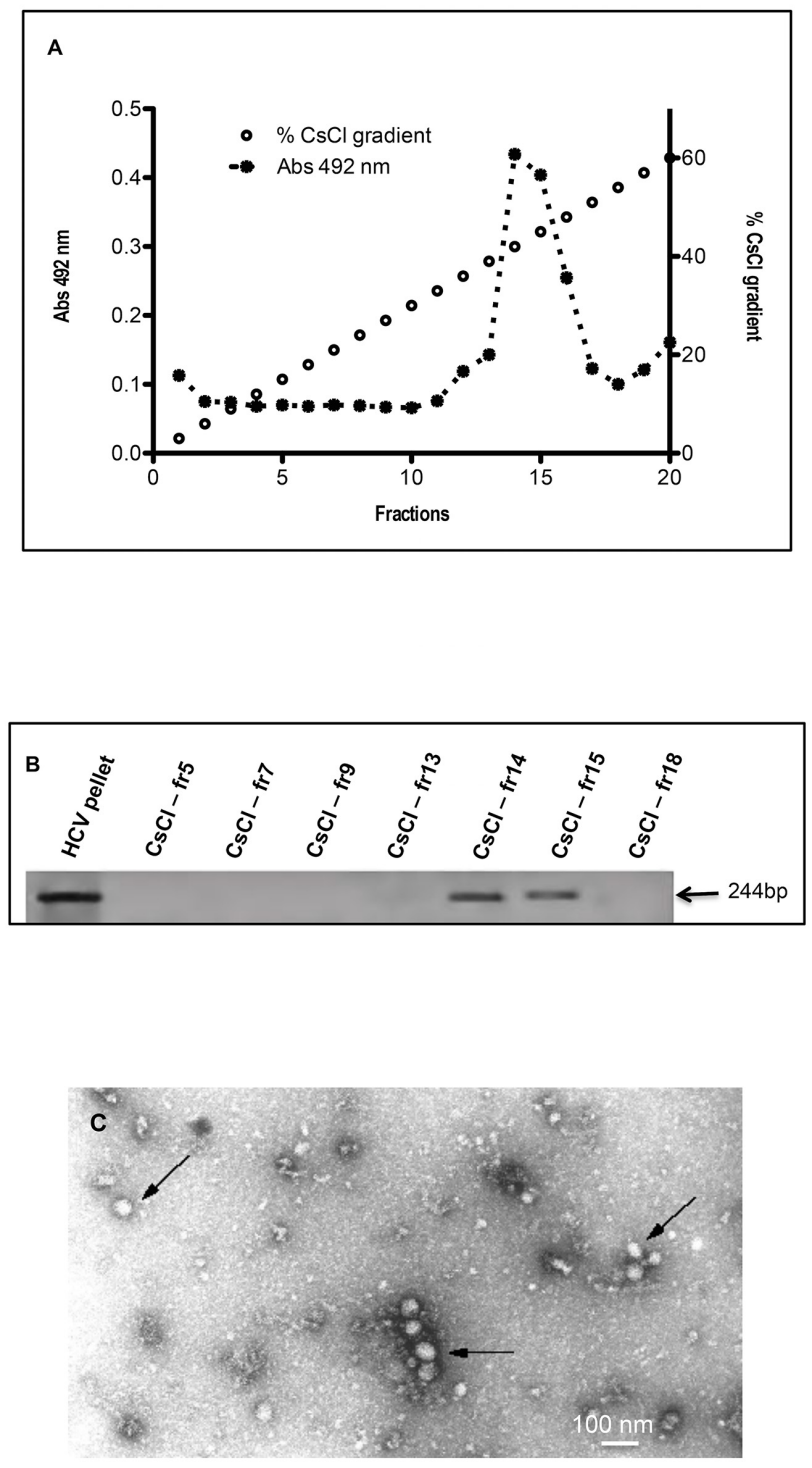
ApoH binds the RNA-containing HCV-related particles. (A) Isolation of HCV-related particles by ultracentrifugation. After the ultracentrifugation at 436,000 x g for 4 h at 4°C of pooled sera from HCV/RNA-positive patients, 100 μL fractions of the pellet were layered onto a 900 μL CsCl gradient ranging from 10% to 60% (w/w) (open dots) and ultracentrifuged again at 300,000 g for 18 h at 4°C. The resulting gradient fractions were ten-fold diluted and tested using the HCV-ApoH immunoassay (black dots). (B) The presence or the absence of HCV-RNA in the centrifugation pellet was revealed by RT-PCR, prior to the CsCl gradient and after the gradient, in some of the resulting gradient fractions (CsCl-fr 5, 7, 9, 13, 14, 15 and 18). (C) CsCl ultracentrifugation gradient fractions corresponding to a density of 1.45 g/mL were layered directly onto ApoH-coated electron microscopy grids to observe the purified HCV-particles as previously done for HBV [44].

Because all the reported studies on the HCV density have been done on the floating lipids, we decided to characterize pelleted HCV particles. Thus, after 1 h of ultracentrifugation, the pellet was further purified on CsCl gradient [10–60% (w/w)]. Ten-fold diluted fractions of this gradient were tested with the ApoH-HCV ELISA immunoassay. Fig 3A shows that the higher HCV/E2 antigen-capture by ApoH-coated plates were identified in fractions 14 to 16, respectively corresponding to the CsCl densities of 1.311 and 1.450 g/mL suggesting that the HCV/E2 antigen recognized by ApoH belongs to a large macromolecular complex. In order to assess whether the HCV-related particles captured by the ApoH-coated ELISA plates contained viral RNA, an open home-made HCV-RT-PCR was performed for some gradient fractions chosen along the shape of the CsCl gradient curve. HCV/RNA was detected in fractions 14 and 15 (Fig 3B). Thus, the viral RNA detection was well correlated with the HCV/E2 immunoassay detection (Fig 3A). To determine the structural features of viral related particles captured by ApoH, electron microscopy analyses were performed with purified particles from the 1.45 g/mL fraction of the CsCl centrifugation gradient. In this fraction, virus-like spherical particles, 55–65 nm in diameter were observed (Fig 3C).

**Fig 4:**
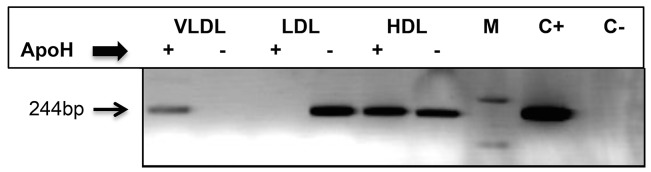
ApoH captures HCV/RNA-containing particles, from low-density and high-density plasma fractions. A pool of 10 HCV/RNA-positive untreated patients was separated into three fractions, respectively corresponding to the floating densities of VLDL, LDL and HDL. One hundred μL of VLDL, 10 μL of LDL and 1 μL of HDL were respectively incubated with 10 μL of ApoH-coated nanomagnetic beads and HCV/RNA was submitted to a home-made HCV RT-PCR. Lanes M, C+ and C- respectively correspond to the DNA molecular mass marker (1 Kb Plus DNA Ladder), the positive control (plasmid pGEM-T easy with an HCV/PCR-insert) and negative PCR control.

Thereafter, we also tried to identify lipoproteic fractions, from HCV/RNA-positive but untreated patients, exhibiting an affinity for ApoH. Thus, a pool (n = 10) of plasma previously used for density analysis was fractionated into four parts, corresponding to the floating densities of VLDL (<1.006 g/L), LDL (1.006 to 1.063 g/L), HDL (1.063 < d < 1.210 g/L) as well as to the bulk plasma proteins. Fig 4 shows the presence of HCV-RNA in VLDL and HDL lipoproteic fractions. In the LDL fraction, HCV-RNA was not detected in the presence of ApoH-beads. Plasma fractions from healthy blood donors remained negative for this test (data not shown). The fact that both HCV RT-PCR and HCV ApoH ELISA tests were positive, confirms that ApoH likely binds to nucleic acid full HCV particles.

### Metal ions influence the ApoH binding to HCV

**Fig 5:**
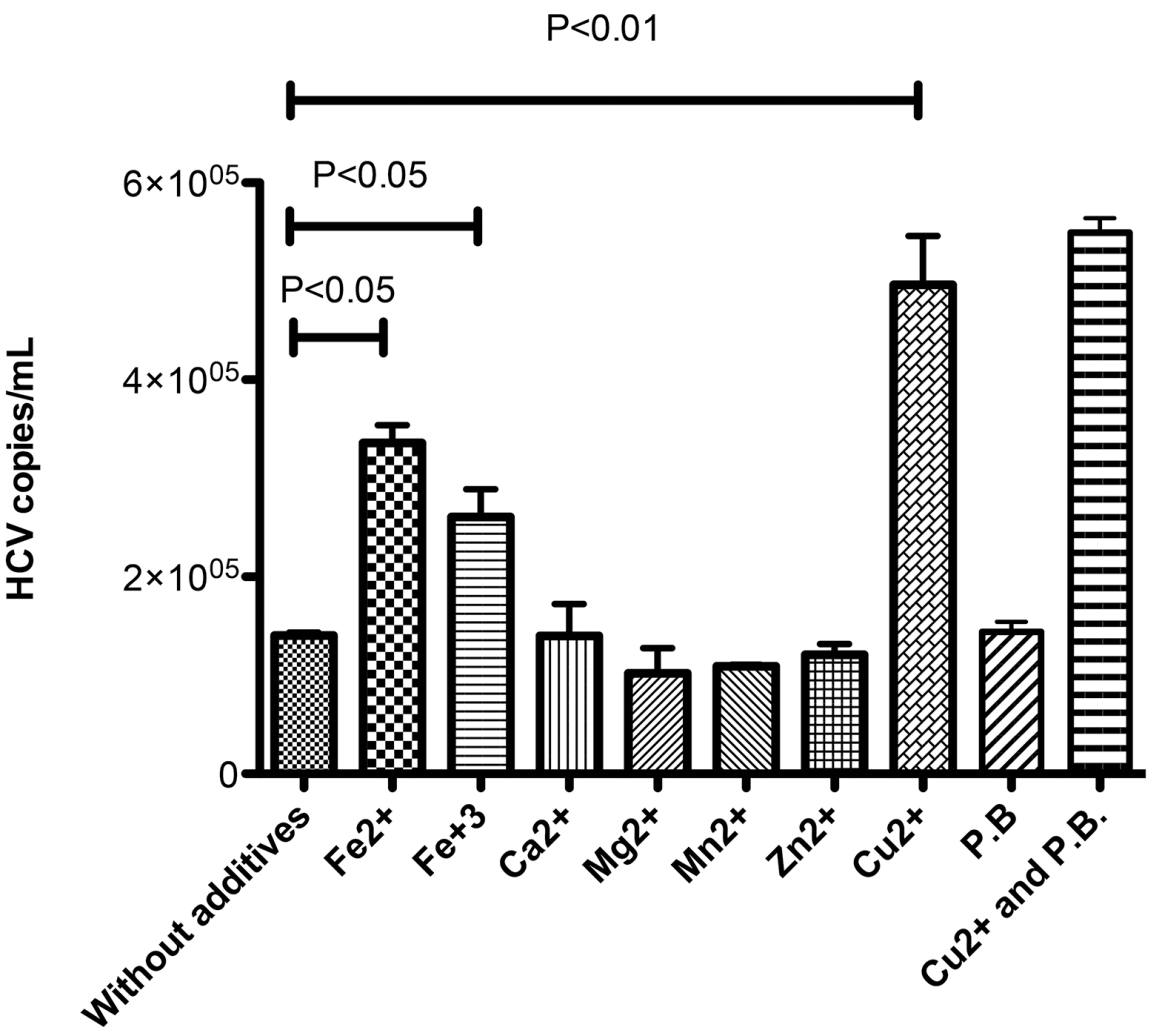
Effect of metal ions or polymyxin B on the HCV-capture by the ApoH system. Sera from two HCV-infected patients were preincubated (or not) for 30 min at 37°C with polymyxin B (P.B., 100 μg/mL) to be subsequently pretreated with different metal ions (2 mmol/L) and with ApoH-coated beads in the conditions stated in the material and method section. Then the ApoH-HCV capture was revealed by an open home-made HCV-RT-qPCR. This experiment represents four similar experiments.

In a previous work [44], we have reported that metal ions influence the HBsAg recognition by ApoH. In order to verify if these metal ions affect the ApoH-HCV binding, we tested the viral capture with ApoH-coated nanomagnetic beads in the presence of different ions, including Fe^2+^, Fe^3+^, Ca^2+^, Mg^2+^, Mn^2+^, Zn^2+^ or Cu^2+^. Fig 5 shows that similar signals were obtained after the addition of Ca^2+^, Mg^2+^, Mn^2+^ or Zn^2+^ into the capture medium. However, the presence of Fe^2+^, Fe^3+^ or Cu^2+^ induced respectively significant three-, two- and five-fold increases of the captured HCV copy numbers.

Iron and copper are redox-active metals and involved in oxidative injury mechanisms, including the lipid peroxidation. To assess whether anionic lipid peroxidation mechanisms are involved in the HCV recognition by ApoH, polymyxin B was added at a concentration of 100 μg/mL. Fig 5 also shows that the addition of polymyxin B does not affects the ApoH binding of HCV, suggesting that this binding does not depend on anionic phospholipids.

### Correlation between the results from post-ApoH-HCV capture home-made qRT-PCR and direct COBAS qRT-PCR assay

**Fig 6:**
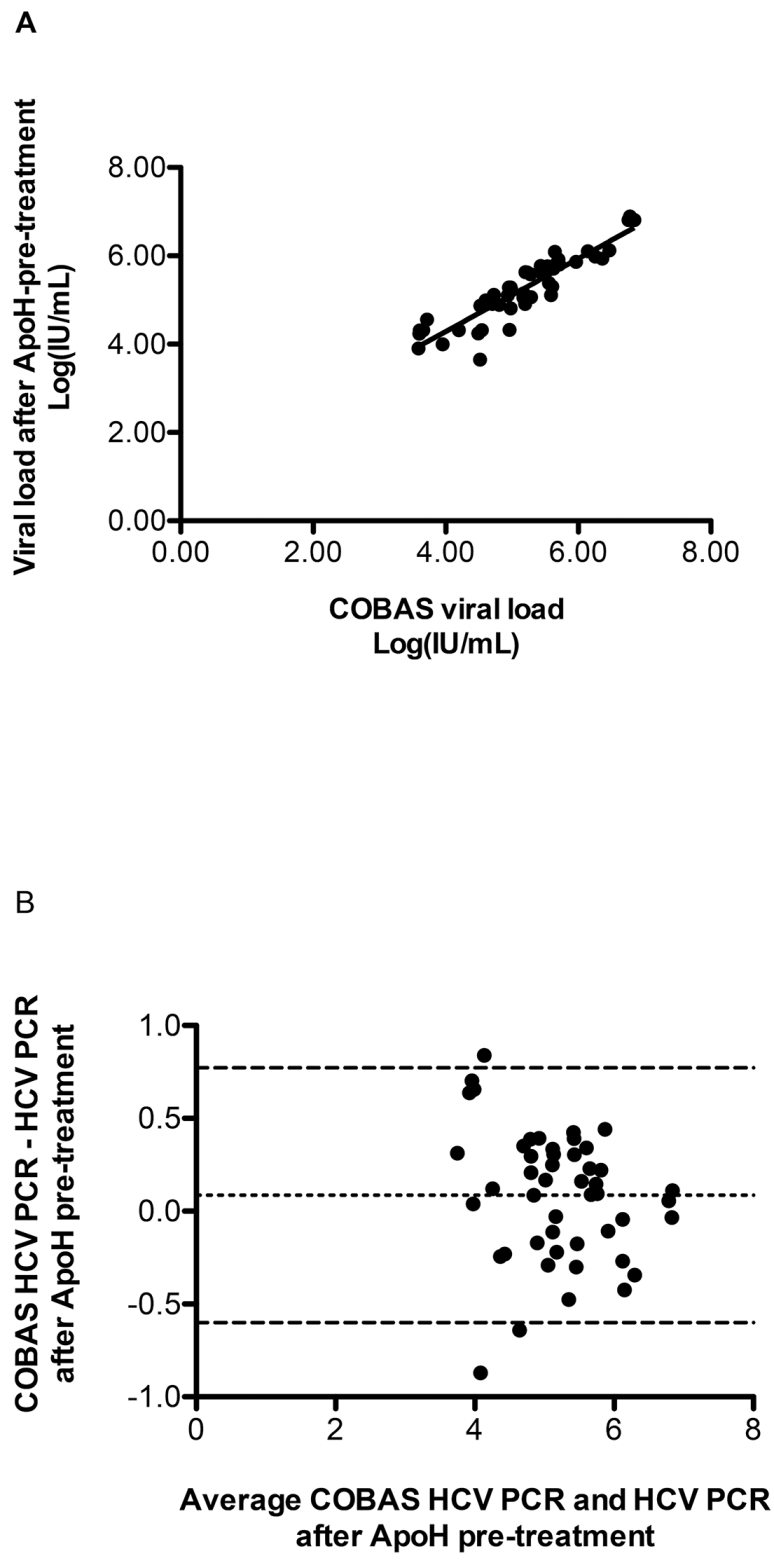
Statistical comparisons and correlations between positive qRT-PCR HCV detection with and without the preparative ApoH-capture HCV step. (A) Scatter-plot comparing the HCV loads using the real-time RT-PCR COBAS® TaqMan® HCV Test, v2.0 alone versus the open home-made HCV real-time RT-PCR assay after the ApoH-HCV capture. Forty-eight clinical samples from HCV-infected patients were tested. The solid line represents the regression curve. (B) Bland-Altman plot depicts the correlation of the viral load figures from COBAS HCV real-time RT-PCR assay alone and the figures resulting from the HCV real-time RT-PCR assay associated with the ApoH sample pretreatment (n = 48). The graph displays a scatter diagram of the differences plotted against the averages of the two measurements. Horizontal lines are drawn at the mean difference and at the mean difference ± 1.96 times the standard deviation, SD, of the differences (95% limits of confidence).

Forty-eight hospital-archived HCV-seropositive samples, with a viral load above 10^3^ IU/mL were processed in parallel using (i) the Real-Time COBAS® TaqMan® HCV Test, v2.0 without the ApoH-sample pretreatment step and (ii) a home-made HCV RT-PCR assay done after the ApoH-sample pretreatment step. A significant correlation was found for both methods (Fig 6A) (r^2^ = 0.8261, P < 0.0001, Pearson’s r = 0.9089, 95% CI = 0.8423–0.9482 for n = 48) for a viral load concentration varying between 10^3^ and 10^7^ IU/mL. The Bland–Altman plot shows the differences between the two assays (Fig 6B). The bias (0.086) to these measures is very close to zero, indicating that the obtained results using both methods generate similar results on plasma with high viral loads, thus confirming their correlation.

Forty seven hospital-archived samples from HCV-seropositive patients, including 22 of them exhibiting a viral load below 10^3^ IU/mL and 25 of them, exhibiting a negative COBAS HCV RT-PCR, were processed to detect or not HCV/RNA after an ApoH-sample pretreatment using ApoH-coated magnetic beads. All the 22 serum samples from HCV-seropositive patients, with previously detected low viral loads, were also found positive with the two-step ApoH-capture and RT-PCR method and some of them exhibited a higher HCV-load. Eleven samples out of 25 previously determined COBAS HCV-RT-PCR negative sera turned RT-PCR HCV-positive after the ApoH-sample pretreatment step. The absence of HCV/RNA detection was confirmed for the remaining 14 samples out of these 25 samples. Obviously, for these samples we didn’t statistically find any correlation between the two methods.

### Enhanced detection of HCV through pre-analytical sample treatment with ApoH-coated nanomagnetic beads

**Fig 7:**
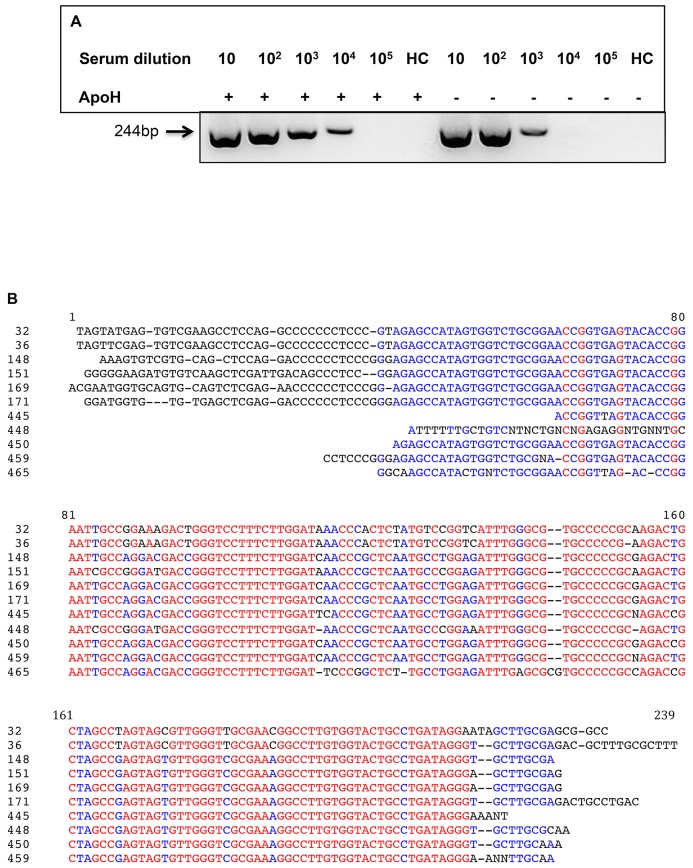
Enhanced detection of HCV following HCV capture with ApoH-coated beads. (A) In the presence or in the absence of the ApoH-coated beads, HCV/RNA from 10-fold serial dilutions of a single HCV-infected patient serum was detected in gel after HCV RT-PCR amplification. HCV-negative healthy control serum (HC). (B) List of HCV sequences from 11 HCV-seropositive patients (samples #: 32, 36, 148, 169, 171, 151, 459, 450, 465, 448, 445) with prior negative COBAS HCV RT-PCR, but tested as HCV-positive with the two-step detection method consecutively including the ApoH-sample pretreatment and the home-made HCV RT-PCR.

To explain both results above, results on viral loads below 10^3^ IU/mL and those of Fig 4 showing that, for 100 μL of VLDL, an HCV-RNA signal was observed only in the presence of ApoH-coated nanomagnetic beads (lane #1 as compared with lane #2), we hypothesized that the HCV capture by ApoH-coated nanomagnetic beads permitted its sensitive detection by a prior viral cleansing and the elimination of PCR inhibitors. Indeed, we previously reported for the cardiopulmonary ANDES hantavirus [56] that, despite a relatively high viral load >5x10^4^ per reaction, viruses were detected only in the presence of a prior ApoH-sample pretreatment. Thus, in this case the sample pretreatment cleansed inhibitors rather than concentrated viruses. This was checked using serially diluted HCV-RNA-positive serum submitted to our home-made HCV RT-PCR in the presence or the absence of ApoH-coated nanomagnetic beads. For this tested single serum, a strong difference of sensitivity was observed between the results obtained in the presence of ApoH-beads and in their absence (Fig 7A). Indeed, in the presence of ApoH-coated beads, the tested serum was yet HCV positive at the 10^4^–fold dilution, while for the same dilution, in the absence of beads, no signal was detected. As noted above, 25-tested HCV-seropositive samples were COBAS HCV-PCR negative. Eleven COBAS HCV-PCR negative sera generated a positive result with the home-made HCV RT-PCR following sample pre-treatment with ApoH-beads. The presence of HCV was confirmed by sequence analysis. Thus, when these sequences (Fig 7B) were compared with several annotated sequences from different nucleic acid sequence banks using the NCBI-Blast sequence comparison software®, it appeared that they exhibited different sequences close to different HCV genotypes (Table 4). Thus, introducing the two-step procedure, we confirmed that ApoH-sample preparation coupled with the home-made HCV RT-PCR was able to detect HCV in 44% of samples, from the archived-hospital collection, that were previously HCV-negative with COBAS HCV RT-PCR.

**Table 4:** Nucleic acid sequence identification of HCV amplicons yielded with the two-step HCV detection procedure including ApoH-virus capture and home-made HCV RT-PCR.

Sample	Blast (Annotated accession numbers)^a^	Identity	e-value
32	gb|AF217306.1|AF217306 Hepatitis C virus clone Sot24 5'UTR sequence	96%	9e-92
36	gb|L34369.1|HPCBCC Hepatitis C virus 5' non-coding region	97%	1e-94
148	emb|LN681401.1| Hepatitis C virus 5'UTR region, genotype 1b	99%	9e-102
151	gb|DQ516083.1| Hepatitis C virus subtype 4d isolate 24 polyprotein gene	98%	4e-85
169	gb|GQ418227.1| Hepatitis C virus genotype 1 isolate 06.10.001 5' UTR	98%	2e-93
171	gb|HM771266.1| Hepatitis C virus isolate AA0828 5' UTR	95%	3e-96
445	dbj|AB523078.1| Hepatitis C virus subtype 1a gene for core protein	99%	6e-67
448	emb|LN681369.1| Hepatitis C virus 5'UTR region, genotype 4a, isolate 3cf	99%	9e-61
450	gb|GQ418214.1| Hepatitis C virus genotype 1 isolate 07.10.030 5' UTR	100%	3e-85
459	gb|KP888622.1| Hepatitis C virus isolate US069 5' UTR	98%	8e-82
465	gb|EU256007.1| Hepatitis C virus subtype 1a isolate HCV-1a/US/BID-V464/2006	91%	3e-60

^a^ HCV sequences present in serum samples from HCV seropositive patients with negative RT-PCR COBAS® TaqMan® HCV Test, v2.0, but tested positive with the two-step ApoH-HCV capture and HCV detection method.

## Discussion

In this study, we have evidenced that the scavenger acute phase protein ApoH was able to capture RNA-containing HCV particles from infected patients’ sera independently of the tested genotypes. This binding was specific as an anti-ApoH MAb was able to almost totally abrogate this interaction, whereas no effect was observed in the presence of α1 acid-glycoprotein, another acute phase plasma protein. When comparing HBV and HCV-positive sera, after ultra-centrifugation at 436,000 x g (Table 2), the presence of ApoH bound to the HCV glycoprotein E2 was dispersed in almost all the ultracentrifuged fractions including in the floating lipid phase, supernatant and pellet, whereas the vast majority of captured HBsAg of HBV was found in the pellet fraction. Thus, HCV exhibited highly heterogeneous density as compared with HBV.

To characterize the nature of HCV particles involved in its binding with ApoH, we performed different analyses, including a CsCl centrifugation gradient, detection of different gradient fractions with the anti-HCV/E2, 3A2C11, MAb, HCV RT-PCR, as well as transmission electron microscopy. HCV particles found *in vivo* are reported exhibiting heterogeneous densities, ranging from 1.06 to 1.25 g/mL [23, 37–40]. These differences were attributed to the presence or the absence of host’s lipoproteins, antibodies bound to the circulating viral particles or circulating RNA-positive HCV nucleocapsids [57, 58] that could mask or interfere with the ApoH binding activity. In order to make a preparative viral purification, we have done an ultracentrifugation round of a pool of infected patients’ sera. After ultracentrifugation, the HCV/E2 glycoprotein was mainly detected in the floating lipids phase as well as in the supernatant and in a lesser extent, in the pellet. Since the lipid-floating phase mainly contains VLDL and that the HCV-VLDL complexes have already been studied, we decided to assess the presence of HCV particles in the pellet.

For the studied sera, the maximal HCV/E2 antigen-captures by ApoH-coated plates were found at the respective densities of 1.311 and 1.450 g/mL. Density amplitudes vary from 1.239 to 1.450 g/mL in function of patient’s sera (data not shown) suggesting that the antigen recognized by ApoH belongs to a large macromolecular complex. High-density molecular complexes containing HCV/RNA have been reported but not always characterized [59]. Some of these high-density HCV have been reported as lipoprotein-free virus [19]. Both RT-PCR and electron microscopy observations on ApoH-coated grids evidenced that these complexes contain HCV/RNA and are mainly composed of spherical particles of 55–65 nm in diameter, thus establishing the binding of HCV particles to ApoH.

The HCV-ApoH capture applied to sequentially ultra-centrifuged plasmas confirmed the presence of HCV as a lipoviral particle, in both VLDL and HDL fractions, but not in the LDL one, thus confirming the association of HCV with different lipoprotein fractions that have been already described [60]. The Polymyxin B nonapeptide, an antibiotic showing a high affinity for anionic phospholipids [61], didn’t interfere with the ApoH-HCV interaction, suggesting that this binding is done in an anionic phospholipid-independent manner, which then differs from the interaction observed between HBV and ApoH [44]. This is in line with the VLDL and HDL composition [62] that do not harbour the anionic phospholipids, such as phosphatidyl serine or cardiolipin, which exhibit an affinity for ApoH.

ApoH binding to phospholipids is reported to depend on their oxidation state [63]. Oxidized forms of LDL-associated ApoH are frequently detected in sera from patients with APS and/or systemic lupus erythematous [64]. Oxidized forms of LDL are also significantly increased in patients with chronic hepatitis C and correlated with the corresponding viral load [51]. HCV causes oxidative stress by a variety of processes [52], including metal unbalance [53]. Oxidant and non-oxidant metal ions, such as iron, copper and zinc differently affect the hepatitis disease. HCV-infected patients display low plasma concentrations of zinc but high concentrations of copper and iron as compared with control subjects [65]. The oxidative potential of copper on LDL has been already reported, and in addition, plasma copper levels correlates with viral load [66, 67]. Iron is essential for cell survival, and its excess or deficiency may lead to disease. Increased amounts of iron appear to facilitate infections and this overload linked to the physiopathology of viral hepatitis has been reported [68, 69]. Iron overload is associated with both the increased DNA damage and the lipid peroxidation in HCV-infected patients [70]. In contrast, zinc has antioxidant properties, and its level in chronic liver disease due to hepatitis C is inversely correlated with viral load [66]. This metal has been described as having inhibitory effect on spontaneous lipid peroxidation [71]. Our results on the effect of metal ions on the ApoH–HCV binding confirmed that oxidative mechanisms are involved in these interactions. Thus, ApoH–HCV binding was increased by the addition of iron and copper, which are also involved in both oxidative injury and lipid peroxidation, whereas zinc seems to have no influence. The interaction between ApoH and HCV does not necessarily involve interactions between ApoH and HCV proteins. Indeed, the presence of ApoH has been described in all major lipoprotein density fractions [72]. Moreover, ApoH binds oxidized phospholipids [63] and specifically binds to oxidized LDLs [73]. Thus, ApoH could bind to HCV particles *via* the phospholipids in function of their configuration and their oxidative status.

Altogether, these data lead us to hypothesize that ApoH could play a pivotal role in the evolution of HCV infection, either favouring or inhibiting the infection. In favour of the facilitating hypothesis it has been reported that lipoprotein receptors such as SR-BI or LDL-R [74] are reported to be involved in the uptake of the HCV-associated lipoproteins into hepatocytes. All lipoprotein receptors are able to bind ApoH [75] and the complex resulting from these interactions could participate in either endocytosis or signal transduction [76]. Thus, ApoH in association with HCV particles, as described for the interaction between HCV and ApoE [66] could facilitate the viral entry *via* several lipoprotein receptors. In favour of the inhibition hypothesis, it has been reported that ApoH is able to activate the lipoprotein lipase [77], which inhibits HCV infection by blocking the virus cell entry [78]. Furthermore, ApoH was found to be associated with a rapid clearance of liposomes *in vivo* [79] suggesting a significant role in the immune clearance of the “non-self” particles, such as HCV. The clearance phenomenon could depend on the presence of free active form of the plasmatic ApoH in the organism resulting in different possible pathological consequences of the infection. To support this hypothesis, new data using liver slices and HCV particles report that ApoH limits the HCV replication, furthermore a correlation was observed between high plasma ApoH-concentrations and improved clinical outcome of HCV-infected patients [80].

Here, we have shown that the ApoH-coated beads can be used as an HCV-capture method to enhance the sensitivity of virus detection, resulting either from an endpoint or a quantitative RT-PCR as shown in Fig 6A and 6B. It is of note that in some samples split in two equal volumes, the initial amount of viruses was higher in the presence of ApoH (these initial viral amounts were not saturating for the ApoH-magnetic beads) as compared with the experiment done in the absence of ApoH. Therefore, most of the time this method is able to reveal the presence of HCV in HCV-seropositive clinical samples with negative COBAS HCV RT-PCR detection. We have processed 95 HCV-seropositive hospital-archived patients’ samples, by using the Real-Time COBAS HCV PCR in the absence of the ApoH-sample pretreatment as well as by using a home-made HCV real-time RT-PCR done after the ApoH-sample pretreatment. For viral loads above 10^3^ IU/mL, a significant correlation was found between both methods. In contrast, for viral loads below 10^3^ IU/mL or negative COBAS HCV RT-PCR samples from hospital-diagnosed patients, it appeared that the ApoH-sample pretreatment significantly improved the sensitivity of HCV detection in a genotype-independent manner. In particular, this ApoH sample pretreatment permits to easily detect HCV in those cases where the sole application of current methods generates erroneous false-negative HCV diagnoses. Thus 11 out of 25 (44%) seropositive samples that were COBAS HCV-PCR-negative were found HCV-positive with the open home-made HCV-PCR following the sample preparation with the ApoH-viral capture method. The presence of HCV/RNA was confirmed by DNA sequencing. The Blast nucleic acid sequences similarities with different annotated data banks showed that these sequences belonged to different HCV genotypes. In order to explain these differences between the presence of HCV in the presence or in the absence of ApoH beads, we have hypothesized that ApoH-coated nanomagnetic beads permitted HCV sensitive detection by a prior viral cleansing and elimination of PCR inhibitors. Different studies have reported the presence of PCR inhibitors in nucleic acids extracts, while others underline the adequacy of sampling and nucleic acids extraction in yields of microbial nucleic acids [81, 82]. Consequently, the sample pretreatment with ApoH could be useful tool to permit a sensitive detection of HCV in samples from patients with very low viral loads as well as for a more accurate manner to monitor the efficiency and management of anti-HCV therapies.
